# Could SCUBE-1 be a marker for subclinical atherosclerosis other than carotid artery intima-media thickness in patients with psoriasis?^☆^

**DOI:** 10.1016/j.abd.2022.08.012

**Published:** 2023-05-15

**Authors:** Havva Hilal Ayvaz Çelik, Mevlüt Serdar Kuyumcu, Fevziye Burcu Şirin, Mehmet Cirit, Selma Korkmaz, İjlal Erturan, Seda Çelik, Mehmet Yıldırım

**Affiliations:** a Department of Dermatology, Faculty of Medicine, Suleyman Demirel University, Isparta, Turkey; b Department of Cardiology, Faculty of Medicine, Suleyman Demirel University, Isparta, Turkey; c Department of Biochemistry, Faculty of Medicine, Suleyman Demirel University, Isparta, Turkey

**Keywords:** Atherosclerosis, Carotid intima-media thickness, Psoriasis

## Abstract

**Background:**

Psoriasis is a chronic inflammatory disease that is associated with many inflammatory conditions such as atherosclerosis, hypertension, among others. SCUBE-1 is a protein that plays a role in angiogenesis.

**Objectives:**

The present study aimed to investigate whether SCUBE-1 could indicate subclinical atherosclerosis in psoriatic patients, and to compare SCUBE-1 levels, measurement of carotid artery ıntima-media thickness (CIMT), and metabolic parameters in psoriasis patients and healthy controls.

**Methods:**

Forty-six patients with psoriasis and 43 healthy controls were included. The severity of the disease was assessed with Psoriasis Area Severity Index (PASI) in the patient group. Levels of SCUBE-1, CRP, lipids, and fasting glucose were measured with the enzyme-linked ımmunosorbent assay (ELISA) method, and CIMT measurements were performed by the same cardiologist.

**Results:**

SCUBE-1 levels and CIMT values were significantly higher in the patient group (for both p < 0.05). Moreover, systolic blood pressure, CRP levels, and waist circumference were higher in the patient group even though both groups had similar BMIs (for all p < 0.05). A positive correlation was found between SCUBE-1 and CIMT values among patients, and multiple regression analyses revealed that SCUBE-1 and CIMT are significantly associated with psoriasis as well.

**Study limitations:**

A low number of participants and not including any other inflammatory marker related to angiogenesis or atherosclerosis such as VEGF, adiponectin are the main limitations of the present study.

**Conclusion:**

Despite the severity of the disease, even in psoriasis patients with mild disease the SCUBE-1 level may be an indicator of subclinical atherosclerosis and indicate the risk of cardiovascular disease in the future.

## Introduction

Psoriasis is a systemic inflammatory disease that is frequently associated with metabolic conditions including cardiovascular diseases, hepatosteatosis, obesity, insulin resistance, diabetes mellitus (DM), hypertension, and hyperlipidemia.^1^

The exact pathogenesis of psoriasis is not fully elucidated, but the enlightened part includes mainly T-cell-mediated inflammation characterized by the activation of T-helper (Th)1, Th17, and Th-22 cells.^2^ These activated cells produce many cytokines including Tumor Necrosis Factor (TNF)-α, Interleukin (IL)-1, IL-17, Vascular Endothelial Growth Factor (VEGF), IL-6, IL-22, IL-23, and Interferon-γ (IFN- γ).^2^, ^3^, ^4^ Moreover, these mediators can lead to systemic inflammation that triggers dysfunction of endovascular endothelia, increased oxidative stress, hypercoagulation, and increased angiogenesis which are also the main components of cardiovascular damage and atherosclerosis.^1^, ^5^

SCUBE-1 (Signal peptideCUB-Epidermal growth factor domain-containing protein-1) is a member of a protein family located on the cell surface ‒ the SCUBE proteins ‒ that can be detected in inflammatory processes and angiogenesis.^6^, ^7^, ^8^ In recent studies, it was determined that the SCUBE-1 protein was detected in vascular endothelial cells and platelets which are known to play crucial roles in the arterial thrombosis process.^9^, ^10^ Additionally, there is a study evaluating serum levels of VEGF, SCUBE-1, and SCUBE-3 in patients with psoriasis, in which the authors stated that SCUBE-1 could be related to the pathogenesis of psoriasis.^11^

carotid artery intima-media wall thickness (CIMT) is the measurement of the carotid artery wall thickness which is evaluated by ultrasound and, even early changes in atherosclerosis can be directly diagnosed by this method.^12^ Some studies reported that psoriatic patients have higher CIMT values than healthy non-psoriatic controls.^13^, ^14^, ^15^ Furthermore, they claimed that psoriasis is an independent risk factor for increased CIMT which shows subclinical atherosclerosis.^13^

The present study aimed to evaluate subclinical atherosclerosis in patients with psoriasis by CIMT measurement, SCUBE-1 levels, and metabolic parameters, also to compare them between psoriatic patients and non-psoriatic healthy control subjects, and by so, to investigate that SCUBE-1 could play a role in both pathogeneses of psoriasis and atherosclerosis, as well. To the best of our knowledge, this is the first study in the literature evaluating both SCUBE-1 levels and CIMT measurement concurrently in psoriatic patients and healthy controls.

## Material and method

This prospective case-control study was reviewed by the Ethics Committee of Suleyman Demirel University Hospital and approved by the report of decision number 370 on 23.12.2019. This study protocol was consistent with the Declaration of Helsinki and informed consent was obtained from each participant.

### Study population

Patients diagnosed with psoriasis histopathologically and/or clinically, aged over 18-under 65, presenting to the dermatology outpatient clinic of Suleyman Demirel University Hospital between January 2020 ‒ January 2021, were considered for this study. Patients with a history of any cardiovascular or cerebrovascular events, active infection and inflammatory systemic or skin diseases other than psoriasis, and regular drug intake for any reason were excluded. Detailed patient histories were taken, skin examinations were performed and the presence of arthritis and/or nail involvement was also examined and noted. Psoriasis Area and Severity Index (PASI) scores^16^ were calculated and according to the PASI scores patients were subdivided into 2 groups: a mild disease with a PASI score ≤ 10, and moderate-severe disease with PASI score >10.

Age and sex-matched healthy volunteers without any inflammatory systemic diseases including cardiovascular and/or neurovascular diseases, inflammatory skin disorders, and regular drug intake for any reason were also enrolled as the control group.

The height, weight, waist circumference, diastolic and systolic blood pressures (BPs), and pulse of all subjects were measured.

### SCUBE and other laboratory tests’ measurements

After 10 hours of fasting, venous blood samples were collected and put into a clot activator serum separator tube and centrifuged at 3000 rpm for 10 min to separate serum specimens. Then, these serum samples were stored at −80°C until analysis. Biochemical tests including fasting blood glucose, Triglyceride (TG), total cholesterol, High-Density Lipoprotein (HDL), Low-Density Lipoprotein (LDL), and C-Reactive Protein (CRP) levels were measured by Beckman Coulter AU 5800 chemistry analyzer (Beckman Coulter, Brea, CA, USA). Serum SCUBE-1 levels were measured by using the sandwich Enzyme-Linked Immunosorbent Assay (ELISA) kit (E-EL-H5405 Elabscience, Wuhan, Hubei Province, China). The sensitivity of the ELISA kit was 0.38 ng/mL. The results were expressed as concentration units.

### CIMT ultrasound measurement

This measurement was examined by the same certified and trained cardiologist. Patients were rested for 10 min and were asked to lie in a supine position during the examination. B-mode ultrasonography of both right and left carotid arteries was performed by the physician with a high-resolution (sensitive to a thickness of 0.01 mm) linear 8 MHz transducer for the Vivid S6 Ultrasound machine.

### Statistical analysis

Data were analyzed using IBM SPSS Statistics 21.0. The values of descriptives were presented as mean ± SD (also median, minimum, and maximum values were given). The categorical variables were presented as frequency and percentage. For analysis of continuous variables across groups first, the distribution characteristics were examined with the Shapiro-Wilk test. Chi-Square analysis was used for categorical and qualitative variables, also the Student’s *t*-test was used for comparisons of normally distributed variables, and the Mann-Whitney *U* test for non-normally distributed variables if two groups existed. The relationship between the parameters was assessed using Pearson’s and Spearman’s correlation and analysis. Univariate and multivariate logistic regression analyses consisted of SCUBE-1, CIMT, and the other variables (Systolic BP, waist circumference, glucose, hs-CRP, TG). Statistical significance was considered at a value of p < 0.05.

## Results

Forty-six patients (19 female and 27 male) with a mean age of 40.3 ± 13.8 years and 43 control (18 female and 25 male) subjects with a mean age of 39.9 ± 11.6 years were included in the study. No significant difference was shown between the two groups regarding mean age and sex (p = 0.890, p = 0.958 respectively). The mean duration of the disease in patients was 12.6 years. The mean PASI score was 12.3 in the patient group. 15 patients had mild, and 31 patients had moderate-severe psoriasis according to PASI scores. Patients had a little higher BMI than control subjects with an insignificant difference (25.9 ± 4.8 kg/m^2^ vs. 24.6 ± 3.2 kg/m^2^; p = 0.154). The mean systolic BP and waist circumference were higher in the patient group, and these differences were significant (p = 0.044, and p = 0.005, respectively). The descriptive features of the participants were given in Table 1.

**Table 1:** Baseline characteristics of the study groups (n = 89)

	Patient group	Control group	p-value
	(n = 46)	(n = 43)	
**Age, years**	40.3 ± 13.8	39.9 ± 11.6	0.890^a^
**BMI, kg/m^2^**	25.9 ± 4.8	24.6 ± 3.2	0.154^a^
**Female, n (%)**	19 (41.3)	18 (41.9)	0.958^b^
**Smoking, n (%)**	25 (54.3)	20 (46.5)	0.460^b^
**Alcohol use, n (%)**	9 (19.6)	13 (30.2)	0.244^b^
**Systolic blood pressure, mmHg**	121.8 ± 18.7	115.1 ± 11.9	**0.044**^c^
**Diastolic blood pressure, mmHg**	77.7 ± 9.7	74.0 ± 9.7	0.070^a^
**Pulse, per minute**	79.2 ± 8.9	76.1 ± 6.5	0.067^a^
**Waist circumference, cm**	94.8 ± 14.2	87.9 ± 7.2	**0.005**^c^

Data are given as mean ± standard deviation, number (n). BMI, Body Mass Index.

The bold values are statistically significant values.

a Independent samples *t*-test.

b Chi-Square.

c p < 0.05.

### Laboratory parameters

Statistically significant differences were shown between patients and healthy controls in terms of serum fasting glucose, TG, and CRP levels (for all p < 0.05). All of these parameters were higher in the patient group (Table 2).

**Table 2:** Laboratory parameters of the study groups (n = 89)

	Patient group	Control group	p-value
	(n = 46)	(n = 43)	
**Glucose, mg/dL**	110.1 ± 31.1	93.4 ± 14.2	**0.021**^a^
**Total cholesterol, mg/dL**	191.3 ± 36.1	183.4 ± 36.5	0.365
**Triglyceride, mg/dL**	141.0 ± 67.0	102.6 ± 46.5	**0.002**^a^
**LDL, mg/dL**	118.3 ± 27.4	110.8 ± 27.7	0.200
**HDL, mg/dL**	47.0 ± 13.1	51.8 ± 13.4	0.088
**Hs-CRP, mg/L**	4.1 ± 3.1	1.8 ± 2.1	**0.002**^a^

Data are given as mean ± standard deviation, number (n). HDL, High Density Lipoprotein; LDL, Low-Density Lipoprotein; Hs-CRP, High-Sensitivity C-Reactive Protein.

The bold values are statistically significant values.

a p < 0.05, Mann-Whitney *U*-test.

### SCUBE-1 levels and CIMT measurements

The mean value of SCUBE-1 in patients was 4.47 ± 2.40 whereas it was 2.66 ± 2.49 in the control group, and this difference was significant (p < 0.001). Moreover, significant differences were found between the groups regarding right, left, and mean CIMT measurements (for all p < 0.001) (Table 3).

**Table 3:** SCUBE-1 levels and carotid intima-media thickness values of the study groups (n = 89)

	Patient group	Control group	p-value
	(n = 46)	(n = 43)	
**SCUBE-1 score**	4.47 ± 2.40	2.66 ± 2.49	**<0.001**^a^
**Right CIMT**	0.81 ± 0.07	0.75 ± 0.04	**<0.001**^a^
**Left CIMT**	0.81 ± 0.08	0.74 ± 0.04	**<0.001**^a^
**Mean CIMT**	0.81 ± 0.07	0.75 ± 0.04	**<0.001**^a^

Data are given as mean ± standard deviation, number (n). CIMT, Carotid Intima-Media Thickness.

The bold values are statistically significant values.

a p < 0.001, Mann-Whitney *U*-test.

However, no significant differences were shown between each of the SCUBE-1 values and CIMT measurements according to the severity of the disease based on PASI scores among the patient group (for all p > 0.05) (Table 4).

**Table 4:** Comparing SCUBE-1 levels, right CIMT, left CIMT, and mean CIMT measurements according to the severity of disease among the patient group (n = 46)

	PASI score ≤ 10	PASI score > 10	p-value
	(n = 16)	(n = 30)	
**SCUBE-1 score**	4.80 ± 2.35	4.28 ± 2.44	0.487
**Right CIMT**	0.82 ± 0.07	0.81 ± 0.06	0.714
**Left CIMT**	0.84 ± 0.08	0.78 ± 0.14	0.138
**Mean CIMT**	0.83 ± 0.07	0.80 ± 0.08	0.153

Data are given as mean ± standard deviation, number (n). CIMT, Carotid Intima-Media Thickness.

p < 0.05, Mann-Whitney *U*-test.

Moreover, SCUBE-1 and CIMT values were positively correlated among the patients (Table 5). A positive correlation was also shown between SCUBE-1 levels and systolic BP, and diastolic BP. In addition, positive correlations were found between systolic and diastolic BPs, BMI and waist circumference, total cholesterol, and, LDL levels (Table 5). Multiple linear regression analyses revealed that the presence of psoriasis was significantly associated with CIMT and SCUBE-1 as well (Table 6).

**Table 5:** Correlation of variables with each other among the patient group (n = 46)

	BMI	Duration of disease	Smoking	Systolic BP	Diastolic BP	Waist circumference	Total cholesterol	LDL	SCUBE1	CIMT	PASI
**BMI**											
PC	1										
Sig. (2-tailed)											
**Duration of disease**											
PC	0.130	1									
Sig. (2-tailed)	0.389										
**Smoking**											
PC	0.013	−0.193	1								
Sig. (2-tailed)	0.906	0.199									
**Systolic BP**											
PC	0.381^a^	−0.043	0.048	1							
Sig. (2-tailed)	0.000^a^	0.778	0.658								
**Diastolic BP**											
PC	0.299^a^	0.171	−0.059	0.661^a^	1						
Sig. (2-tailed)	0.004^a^	0.255	0.585	0.000^a^							
**Waist circumference**											
PC	0.676^a^	0.213	0.079	0.404^a^	0.330^a^	1					
Sig. (2-tailed)	0.000^a^	0.155	0.464	0.000^a^	0.002^a^						
**Total cholesterol**											
PC	0.213^b^	0.028	0.010	0.198	0.097	0.166	1				
Sig. (2-tailed)	0.045^b^	0.855	0.925	0.063	0.365	0.120					
**LDL**											
PC	0.222^b^	0.019	0.067	0.177	0.127	0.151	0.923^a^	1			
Sig. (2-tailed)	0.037^b^	0.902	0.532	0.097	0.235	0.158	0.000^a^				
**SCUBE-1**											
PC	0.135	−0.204	0.091	0.294^a^	0.316^a^	0.205	0.156	0.203	1		
Sig. (2-tailed)	0.208	0.175	0.398	0.005^a^	0.003^a^	0.053	0.146	0.057			
**CIMT**											
PC	−0.037	0.109	0.019	0.363^a^	0.272^a^	0.055	0.144	0.203	0.286^a^	1	
Sig. (2-tailed)	0.727	0.472	0.856	0.000^a^	0.010^a^	0.611	0.179	0.057	0.007^a^		
**PASI**											
PC	0.171	0.130	0.198	0.079	0.097	0.010	−0.059	−0.193	0.028	0.144	1
Sig. (2-tailed)	0.255	0.389	0.063	0.464	0.365	0.925	0.585	0.199	0.855	0.357	

BMI, Body Mass Index; BP, Blood Pressure; LDL, Low-Density Lipoprotein; CIMT, Carotid Intima-Media Thickness; PC, Pearson Correlation.

a Correlation significant at 0.01 level (2-tailed).

b Correlation significant at 0.05 level (2-tailed).

**Table 6:** Univariable and multivariable regression analyses of parameters in psoriasis

	Univariable	p‒value	Multivariable	p-value
	OR (95% Cl)		OR (95% Cl)	
**Systolic blood pressure**	0.970 (0.941‒1.000)	0.052		
**Waist circumference**	0.947 (0.909‒0.986)	**0.008**^a^	0.968 (0.919‒1.019)	0.212
**Glucose level**	0.965 (0.936‒0.994)	**0.019**^a^	0.996 (0.969‒1.023)	0.764
**Hs-CRP**	0.733 (0.579‒0.927)	**0.010**^a^	0.848 (0.649‒1.107)	0.225
**Triglyceride**	0.988 (0.980‒0.996)	**0.005**^a^	0.992 (0.981‒1.003)	0.155
**CIMT**	0.960 (0.940‒0.981)	**<0.001**^b^	0.965 (0.945‒0.976)	**0.003**^a^
**SCUBE-1**	0.729 (0.598‒0.888)	**0.002**^a^	0.828 (0.652‒1.051)	**0.031**^a^

CI, Confidence Interval; OR, Odds Ratio; Hs-CRP, High-Sensitivity C-reactive protein; CIMT, Carotid Intima-Media Thickness.

The bold values are statistically significant values.

a p < 0.05.

b p < 0.001.

## Discussion

Evidence suggests that beneath the inflammation, angiogenesis and endothelial dysfunction are also significant components of the pathogenic mechanisms in psoriasis.^17^, ^18^ The association between psoriasis and increased risk of cardiovascular diseases has been proven in many studies^17^, ^19^, ^20^, ^21^, ^22^ but the underlying mechanisms remain elusive. There are two proposed underlying mechanisms: First, cardiovascular risk factors in the limelight (DM, insulin resistance, hypertension, altered lipid profiles, high BMI, smoking) can be seen more in psoriatic patients compared to the rest of the population;^23^, ^24^ the second one is psoriasis itself which systemic inflammation may also directly affect vessel walls and as a result inflamed vessels cause a higher risk of cardiovascular diseases and mortality.^25^ In a population-based study, the authors showed that psoriasis was associated with increased mean CIMT, but not with carotid plaque prevalence.^26^ In another study from Italy, a significant increase in mean CIMT values was seen in patients with psoriasis, also a strong positive correlation between CIMT and PASI scores was shown.^27^ Similar to the literature, the authors showed a significant difference in CIMT values of psoriasis patients than healthy controls. Furthermore, systolic and diastolic BPs were significantly higher in the patient group whereas both groups did not have any difference in terms of smoking and alcohol habits, and BMI. In addition, contrary to the study mentioned before, the authors did not find either significant differences in CIMT values according to the severity of the disease, nor a correlation between CIMT values and PASI scores. The present results showed that even patients with mild disease may have a higher risk of the presence of subclinical atherosclerosis, and in our subjects, the possible underlying mechanism causing this difference is thought to be related to the disease itself.

SCUBE-1 belongs to the SCUBE proteins family which are increased in inflammatory processes and hypoxia-associated conditions, and are thought to have a role in both physiological and pathological angiogenesis.^28^ In the literature, expression of SCUBE-1 was shown in especially vascular endothelial cells of highly vascular tissues,^8^ and SCUBE-1 levels were found to increase in conditions such as acute coronary syndrome, acute ischemic stroke, DM, malignities, and also psoriasis.^9^, ^10^, ^11^, ^29^, ^30^ Furthermore, the SCUBE-1 levels were decreased by treatments against IL-1β and TNF-α.^8^ Till now, there is only one study evaluating SCUBE-1 in patients with psoriasis, and in this study, the levels of VEGF, SCUBE-1, and SCUBE-3 were reported significantly different between patients with psoriasis and healthy controls.^12^ The authors also reported that SCUBE-1 had 83% sensitivity and 62% specificity for predicting psoriasis. Thus, they suggested that the SCUBE protein family may be a potential marker in the process and screening of pathological angiogenesis in psoriatic patients. Similarly, the authors showed significantly elevated SCUBE-1 levels in the patient group than in healthy controls. In addition, multiple regression analysis and correlation results between CIMT and SCUBE-1 indicated that SCUBE-1 is strongly associated with atherosclerosis, and could be a useful and independent biomarker that shows subclinical atherosclerosis besides CIMT measurement.

In addition to these, CRP is an acute-phase response protein that is involved in many inflammatory processes such as angiogenesis, thrombosis, and activation of the complement system.^31^ Elevated CRP levels were shown related to the risk of future cardiovascular events.^32^ In the present study, the authors showed significantly increased CRP levels in the patient group which may show us a higher prevalence of underlying atherosclerosis and cardiovascular disorders in psoriasis patients. Waist circumference is a clinical marker of obesity that is associated with atherosclerosis and cardiovascular disorders. Moreover, it is assumed as an independent noninvasive measurement of subclinical atherosclerosis.^33^ In the present study, although the patients had similar BMI to the healthy controls, the authors found the waist circumference measurement to be significantly higher in the patient group. The authors also showed a positive correlation between waist circumference and BPs. These results suggest that waist circumference measurement is also an important value showing increased BP, and possible subclinical atherosclerosis even if patients are not obese.

## Conclusion

Although the present study has some limitations such as a low number of participants, and not including any other inflammatory marker related to angiogenesis or atherosclerosis such as VEGF, adiponectin, among others; there has been no other study evaluating metabolic parameters, serum SCUBE-1, and CIMT concurrently in patients with psoriasis in the literature. With these results, the authors showed that besides CIMT measurement, SCUBE-1 might be an important and independent marker to show subclinical atherosclerosis via participation in the pathological angiogenesis process in patients with psoriasis. Moreover, serum CRP levels and waist circumference could be particularly related to subclinical atherosclerosis and may be indicators of future cardiovascular diseases. This pioneering study should be verified by prospective longitudinal future studies with larger sample sizes and more inflammatory markers. The authors think that in the future when the pathways and mechanisms that SCUBE-1 plays a role in the pathogenesis of psoriasis are better understood, it could be a potential target for future therapeutics especially to prevent underlying pathological angiogenesis and subclinical atherosclerosis in psoriasis.

## Financial support

None declared.

## Authors’ contributions

This manuscript has been read and approved by all the authors and has not been published.

Havva Hilal Ayvaz Çelik: Approval of the final version of the manuscript; critical literature review; data collection, analysis and interpretation; effective participation in research orientation; ıntellectual participation in propaedeutic and/or therapeutic; management of studied cases; preparation and writing of the manuscript.

Mevlüt Serdar Kuyumcu: Approval of the final version of the manuscript; critical literature review; data collection, analysis and interpretation; effective participation in research orientation; ıntellectual participation in propaedeutic and/or therapeutic; ıntellectual participation in propaedeutic and/or therapeutic; management of studied cases; manuscript critical review; statistical analysis; study conception and planning.

Fevziye Burcu Şirin: Approval of the final version of the manuscript; data collection, analysis and interpretation; manuscript critical review; study conception and planning.

Mehmet Cirit: Data collection, analysis and interpretation; effective participation in research orientation.

Selma Korkmaz: Manuscript critical review.

İjlal Erturan: Manuscript critical review.

Seda Çelik: Data collection, analysis and interpretation.

Mehmet Yıldırım: Manuscript critical review.

## Conflicts of interest

None declared.
